# Primary thyroid MALT lymphoma with a probable brain secondary in a male patient: A case report

**DOI:** 10.1016/j.ijscr.2023.109126

**Published:** 2023-12-07

**Authors:** B.M. Munasinghe, C.T. Karunatileke, J. Prashanthan, N.D. Ranathunga

**Affiliations:** a Department of Anaesthesiology and Intensive Care, Kent and Canterbury Hospital, Canterbury CT1 3NG, UK; b Department of Surgery, District General Hospital, Mannar, Sri Lanka; c Department of Anaesthesiology and Intensive Care, District General Hospital, Mannar, Sri Lanka; d Department of Histopathology, District General Hospital, Mannar, Sri Lanka

**Keywords:** MALT lymphoma, Thyroid, Hashimoto thyroiditis, Prognosis

## Abstract

**Introduction and importance:**

Primary Mucosa-associated lymphoid tissue (MALT) lymphoma of the thyroid is a rare tumor.

**Presentation of case:**

A previously well male in his 50s presented to our institution with difficulty in breathing and sleep apnea. He was diagnosed with a large retrosternal multinodular goiter with level 2 unilateral cervical lymphadenopathy. Fine needle aspiration cytology of the thyroid revealed chronic thyroiditis and the enlarged lymph node cytology was inconclusive. He underwent total thyroidectomy and level VI bilateral cervical lymph node clearance. The histology revealed an extra-nodal marginal zone lymphoma of MALT. A whole-body CT scan did not demonstrate any other primary site. The patient received 4 cycles of local radiotherapy. Subsequently, he was diagnosed with a brain tumor not amenable to surgical interventions following persistent headaches. He died shortly after due to complications of probable cerebral metastasis.

**Case discussion:**

MALT lymphomas of the thyroid carry a good prognosis; however, no universal guidance exists regarding the optimal therapy and follow-up.

**Conclusion:**

This case report highlights the importance of early diagnosis, identification of poor prognostic factors, and patient-tailored therapy and follow-up.

## Highlights

• MALT lymphoma of the thyroid could present as rapid growing tumours.
• Chronic thyroiditis is a common association.
• Metastasis is unusual in MALT lymphomas of the thyroid.
• The described is a rare case of an advanced tumor with probable brain metastasis.
• Long-term follow-up is required to detect recurrence and initiate therapy early.

## 1 Introduction

Extra-nodal marginal zone lymphoma of mucosa-associated lymphoid tissue (MALT) in the thyroid gland is a rare, slow-growing malignant tumor accounting for 1–2 % of extranodal lymphomas [1]. Thyroid MALT lymphomas are B-cell derived and predominantly occur in the background of chronic inflammation, mainly Hashimoto thyroiditis [2]. Most patients remain asymptomatic and surgery is warranted when pressure symptoms occur or the histopathology is inconclusive [3]. Treatment includes surgery in the form of thyroidectomy, neck dissection, and adjuvant radiotherapy or chemotherapy. The prognosis is favorable compared to diffuse large B cell lymphoma, the commonest lymphoma-variant of the thyroid [2,4]. We present a case of a MALT lymphoma of the thyroid in a middle-aged male who succumbed following a probable brain secondary. The case is reported in line with the SCARE criteria [5].

## 2 Case presentation

A previously well South Asian male in his 50s presented to the surgical clinic of our institution, a District General Hospital, with an anterior neck swelling for 5 years with a recent rapid enlargement and associated difficulty in breathing, sleep apnea, and hoarseness of voice. He was euthyroid clinically. There was no history of radiation exposure and no family history of thyroid disorders. A large multinodular goiter (World Health Organization grade 4) with retrosternal extension was diagnosed by clinical examination. His body mass index was 30 kg m^−2^. Airway assessment revealed a mallampati grade of 4 which indicated difficulty.

## 3 Investigations

X-ray neck showed mild tracheal compression and mild tracheal deviation. Ultrasound of the neck confirmed a large multinodular goiter with features of thyroiditis and areas of calcification. A 2 cm retrosternal extension was also noted with level 2 cervical lymphadenopathy on the left side. The thyroid profile was in the normal range. Fine needle aspiration cytology of the thyroid gland suggested chronic thyroiditis and was inconclusive for cervical lymph nodes. A computed tomography of the neck was not performed due to the unavailability in our center and the long waiting list for a patient with worsening obstructive symptoms. An early surgery was planned. Preoperative vocal cord assessment was normal.

**Fig. 1:**
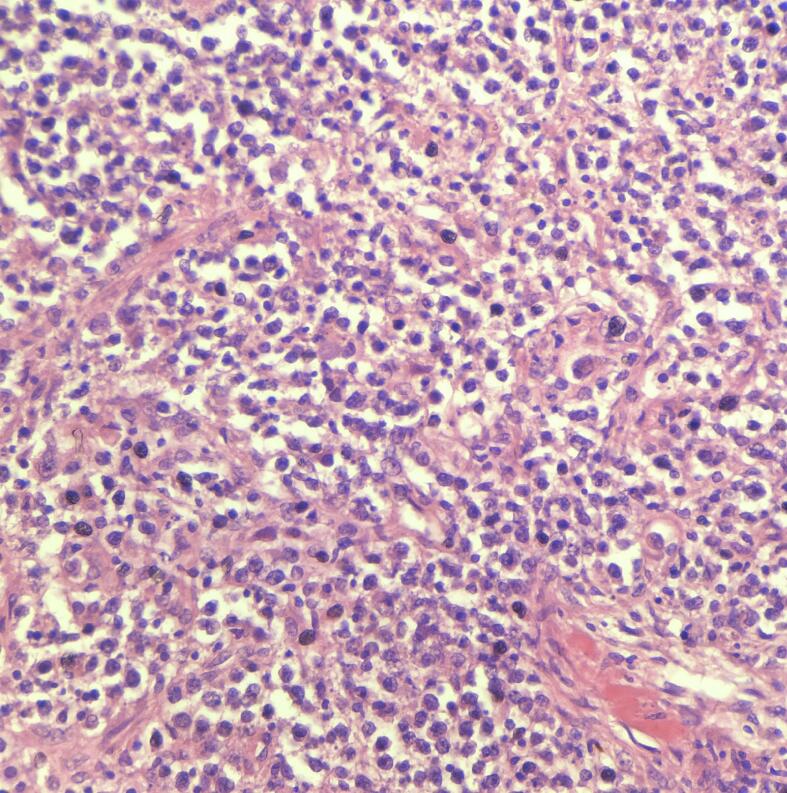
Lymphoepithelial lesions H&E x40.

**Fig. 2:**
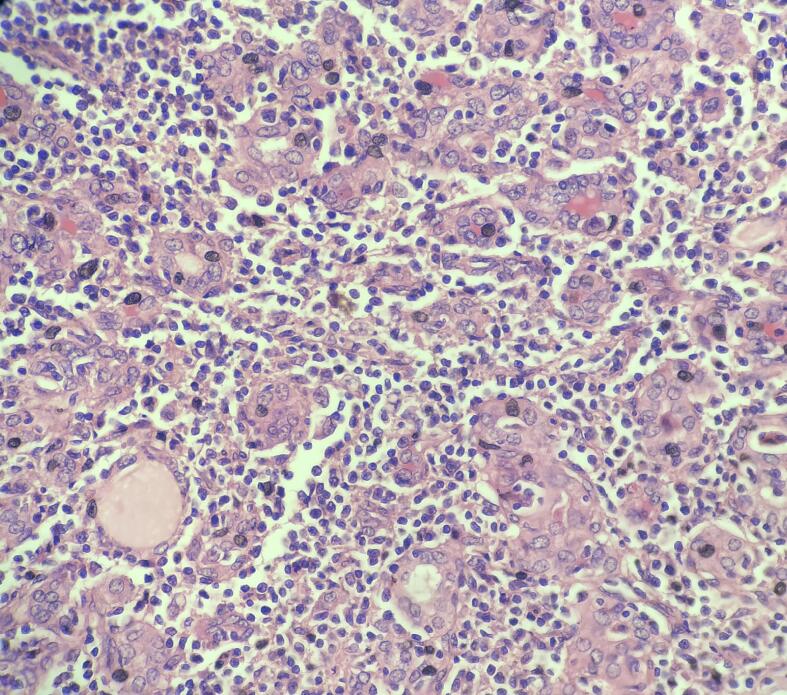
Back ground thyroiditis H&E x10.

Histology revealed diffuse infiltration of a polymorphic population of small to medium size lymphoid cells. Some cells were plasmacytoid. Lymphoepithelial lesions were evident (Fig. 1). Mitoses were sparse. There were no diffuse areas of large cells to suggest transformation into diffuse large B cell lymphoma. A limited IHC panel was performed due to financial restraints. Neoplastic lymphoid cells were positive for CD20 and negative for CD3. confirming the B cell origin. Ki67 index was around 5 %, confirming the low-grade nature of the lymphoma. Therefore, immunohistochemically this was confirmed as a low-grade B cell non-Hodgkin lymphoma, together with morphology that arrived at MALT lymphoma diagnosis. The rest of the thyroid showed chronic autoimmune thyroiditis (Fig. 2). The whole-body CT study was negative for any other tumor sites. The CT head performed later for the persistent headaches revealed a new metastatic deposit.

## 4 Differential diagnosis

MALT lymphoma of the thyroid in the background of chronic autoimmune thyroiditis with subsequent probable metastasis into the brain.

## 5 Treatment

For the initial thyroidectomy, an awake fiberoptic intubation was performed. A wide collar incision was made to gain adequate exposure. During the surgery, both recurrent laryngeal nerves were preserved and a total thyroidectomy was performed. The patient was admitted to the intensive treatment unit and was ventilated to allow the airway oedema to settle. He was extubated on day 02 and discharged home with oral thyroxine and calcium replacement with 1 alpha cholecalciferol.

He was referred to the oncologist following the diagnosis of MALT lymphoma. Localized radiotherapy was commenced in the absence of distant tumor sites. At the end of the fourth cycle of radiotherapy, he was further investigated for new onset, severe headache with pupil inequality. The metastatic deposit of the brain was not amenable to surgical correction.

## 6 Outcome and follow-up

He died shortly after complications of tumor growth in the brain.

## 7 Discussion

MALT lymphoma of the thyroid is a type of primary thyroid lymphoma. These occur more commonly in females (male: female 1:4) of old age (mean age of diagnosis 65 years). [[6], [7], [8]]. Around 90 % of patients have underlying Hashimoto thyroiditis [9]. The studies have shown a 40- to 80-fold increase in the risk of MALT lymphoma in the background of Hashimoto thyroiditis [10]. Diagnosis of MALT lymphoma of the thyroid using cytology and histology is challenging as there can be simultaneous reactive and neoplastic tissue changes [3,11]. Clinically the classical ‘B’ symptoms seen in other lymphomas are also uncommon (around 10 %) in MALT lymphomas of the thyroid [12,13]. A rapid, non-tender enlargement of the thyroid can be the presenting symptom while around 30 % may have compressive symptoms due to mass effect [14,15].

Diagnosis of MALT lymphoma of the thyroid using fine needle aspiration cytology carries a low sensitivity specially in early disease [2,15] and may illustrate chronic thyroiditis in the case of associated Hashimoto thyroiditis. Most recent systematic reviews point out the low sensitivity and specificity of ultrasound in making accurate diagnosis [2]. Definite diagnosis is thus made by histopathological analysis of surgical resection specimens [16]. Further immunohistochemistry studies that yield CD20, Bcl-2, immunoglobulin light chain, and absence of CD5, CD10, and CD23 aid the diagnosis [17]. Co-existing other types of thyroid malignancies (follicular, papillary, and anaplastic) may also be diagnosed using immunohistochemistry. The unavailability of such sophisticated tests in developing countries poses an obstacle and histological analysis following surgical resection of the thyroid for symptomatic goiters may provide an incidental diagnosis [18].

The staging of thyroid MALT lymphomas is still not definite as stage 1E (thyroid only) and 11E (thyroid and regional lymph nodes) cannot be differentiated, however, modified classification systems have been studied [19]. Still, no universal guidance exists with regard to treatment. Open biopsy and isolated-site radiotherapy (OB-ISRT), curative resections, and adjuvant radiotherapy or chemotherapy are all practiced depending on the extent of the disease.

MALT lymphoma of the thyroid has an excellent prognosis (5-year overall survival of 94 % and event-free survival rate of 92 %) [20]. Despite the rarity of both the disease and relapse rates, extended follow-up is recommended by some authors [21,22]. MALT lymphoma usually has an indolent course, and it is quite unusual to metastasize to brain early. The brain tumor in our patient is most likely a secondary from the thyroid tumor considering the temporal association of the events; however, this could not be proven definitely as no histology of the brain lesion was performed.

## Consent

Written informed consent was obtained from the next-of-kin of the patient for the publication of this case report and accompanying images. A copy of the written consent is available for review by the Editor-in-Chief of this journal on request.

## Provenance and peer review

Not commissioned, externally peer-reviewed.

## Ethical approval

Our institution does not require ethical approval for reporting individual cases or case series.

## Funding

This research did not receive any specific grant from funding agencies in the public, commercial, or not-for-profit sectors.

## Author contribution

Clinical management, Concept, consent, literature review, drafting of the initial and final manuscript - **BM, CK**. **JP, ND**.

Approval of the final manuscript- **All authors**.

## Guarantor

B.M. Munasinghe.

## Research registration number

Not applicable.

## Conflict of interest statement

None declared.
